# From Severity to Surgical Load: Long-Term Burden of Care in Cleft Lip Patients

**DOI:** 10.3390/dj14060376

**Published:** 2026-06-17

**Authors:** Ivan Ginev, Youri Anastassov, Kostadin Gigov, Petra Kavradzhieva

**Affiliations:** 1 Department of Propedeutics of Surgical Diseases, Section of Plastic, Reconstructive and Aesthetic Surgery and Thermal Trauma, Faculty of Medicine, Medical University of Plovdiv, 4002 Plovdiv, Bulgaria; yanastassov@gmail.com (Y.A.); kavradjieva@gmail.com (P.K.); 2 “St. George” University Hospital, “Peshtersko Shosse” Blvd. 66, 4002 Plovdiv, Bulgaria

**Keywords:** cleft lip, surgical burden, long-term follow-up

## Abstract

**Background**: Cleft lip with or without cleft palate (CL ± P) is a common congenital anomaly requiring a multidisciplinary team approach from birth into adulthood. Many patients undergo multiple secondary procedures on the lip, nose, and alveolus, representing a substantial long-term “burden of care” for families and health systems. The relationship between preoperative cleft severity and the cumulative number of surgical interventions into late adolescence remains insufficiently characterized. **Methods**: A retrospective cohort study was conducted on 166 patients with cleft lip ± cleft palate treated at a single tertiary cleft center. All patients underwent primary cheiloplasty, with or without concomitant gingivoperiosteoplasty (GPP), and had follow-up extending to a mean age of 18 years. Preoperative nasolabial deformity was graded into four categories (mild, moderate, severe, and very severe) using standardized photographic assessment. The primary outcome was the total number of cleft-related surgical interventions on the lip, nose, and alveolus, including the primary operation and all subsequent corrective procedures. Associations between preoperative severity and surgery counts were analyzed using the Kruskal–Wallis test and Bonferroni-adjusted pairwise comparisons. **Results:** All 166 patients underwent a primary procedure, either cheiloplasty alone (*n* = 86; 51.8%) or cheiloplasty combined with GPP (*n* = 80; 48.2%). A second surgical intervention was performed in 111 patients (66.8%), yielding 138 procedures, most commonly GPP with bone grafting (*n* = 54), corrective cheiloplasty (*n* = 48), GPP without graft (*n* = 23), and rhinoplasty (*n* = 12). A third intervention was performed in 48 patients (28.9%; 70 procedures), predominantly rhinoplasty and additional cheiloplasties, and a fourth intervention in 13 patients (7.8%; 17 procedures), mostly staged rhinoplasty and lip revisions. Overall, 56 patients (33.7%) had only one (primary) operation, 50 (30.1%) had two, 27 (16.3%) had three, 18 (10.8%) had four, 13 (7.8%) had five, and one patient (0.6%) had six surgical interventions. The total number of operations differed significantly across severity grades (Kruskal–Wallis *p* < 0.001). Patients with mild and moderate severity had significantly fewer surgeries than those with severe or very severe deformities (all *p* ≤ 0.023), whereas differences between mild vs. moderate and severe vs. very severe were not significant. **Conclusions**: In this cohort of patients with cleft lip followed to a mean age of 18 years, two-thirds required at least one secondary procedure, and nearly one-fifth underwent four or more surgeries. Higher preoperative severity was strongly associated with greater surgical burden, particularly when comparing mild or moderate deformities to severe and very severe clefts. These findings underline the importance of preoperative severity assessment for family counseling, expectation management, and the design of treatment protocols aimed at minimizing the long-term burden of care while preserving functional and esthetic outcomes.

## 1. Introduction

Cleft lip with or without cleft palate (CL ± P) is among the most common congenital craniofacial anomalies worldwide, with an estimated prevalence of approximately 1 in 700 to 1 in 1000 live births, varying by population and cleft subtype [1]. Management typically spans from early infancy to adulthood and requires coordinated input from surgeons, orthodontists, maxillofacial surgeons, otolaryngologists, speech therapists, pediatric dentists, and psychologists. Over recent decades, refinements in surgical techniques and multidisciplinary protocols have substantially improved outcomes [2,3,4,5,6,7].

The concept of “burden of care” has gained prominence as a way to capture the cumulative impact of this lengthy treatment trajectory on patients, families, and health systems [5,6,7,8,9]. In cleft care, burden of care encompasses repeated hospital visits, numerous staged operations and anesthetic exposures, travel and time costs, and psychosocial strain [5,6,7]. This study focuses on the importance of cleft severity in the lip–nose–alveolus region, as palatal cleft severity appears to be less closely related to surgical burden, being more directly determined by the surface area involved.

Several cohort and database studies have quantified the number of cleft-related procedures from birth to adolescence or early adulthood, often reporting that children with CL ± P undergo an average of 5–10 surgical procedures, particularly those with complete cleft lip and palate or bilateral deformities. Centralized, protocol-driven care in accredited cleft centers appears to reduce the early surgical burden compared with fragmented treatment in non-specialized settings [7,8,9,10].

Anatomic severity of the initial cleft-lip–nasal deformity is increasingly recognized as a key determinant of both functional and esthetic outcomes. Objective severity indices for unilateral and bilateral cleft lip include measures such as lip height deficiency, horizontal lip gap, columellar deviation, nostril asymmetry, and alveolar segment displacement. These indices show high inter-rater reliability and correlate with surgeons’ subjective assessments of deformity. Long-term follow-up series of patients with complete bilateral cleft lip and palate (BCLP), complete cleft lip and alveolus (CLA), or unilateral CL ± P report that more severe baseline deformity is associated with greater need for revisional lip and nasal surgery and higher rates of orthognathic procedures [10,11,12,13,14,15,16]. However, only limited work has explicitly linked preoperative severity grades to the total number of cleft-related operations on the lip, alveolus, and nose across childhood, adolescence, and young adulthood [1,2]. The present study provides a more detailed analysis of the average number of surgical interventions performed for each cleft severity category.

Adult and late adolescent outcome studies show that many individuals with CL ± P still seek or require further operations in their teens and twenties, including rhinoplasty, lip revision, orthognathic surgery, and alveolar reconstruction, to address persistent functional and esthetic issues [10,11,12,13,14,15,16]. The need for long-term follow-up and assessment of the surgical burden in patients aged 18 years or older is therefore well recognized, and this study aims to address this gap.

In addition, patient-reported outcome measures (PROMs) such as the CLEFT-Q have demonstrated that late surgeries can meaningfully improve quality of life [14,15,16,17,18,19,20,21,22]. Together, these data emphasize that cleft lip and palate are life-long conditions whose treatment extends well beyond primary repair.

The present retrospective cohort study aims to address a specific gap in the literature by examining how the preoperative severity of the cleft-lip–nasal deformity relates to the long-term surgical burden of care in patients followed to a mean age of 18 years. The objectives were to describe the distribution and timing of primary and secondary operations on the lip, nose, and alveolus; to analyze the association between preoperative severity category and the total number of cleft-related operations and to identify high-severity subgroups that may benefit from tailored treatment strategies and enhanced counseling [5,6,7,8,9,15,16,17].

## 2. Materials and Methods

**Figure 1:**
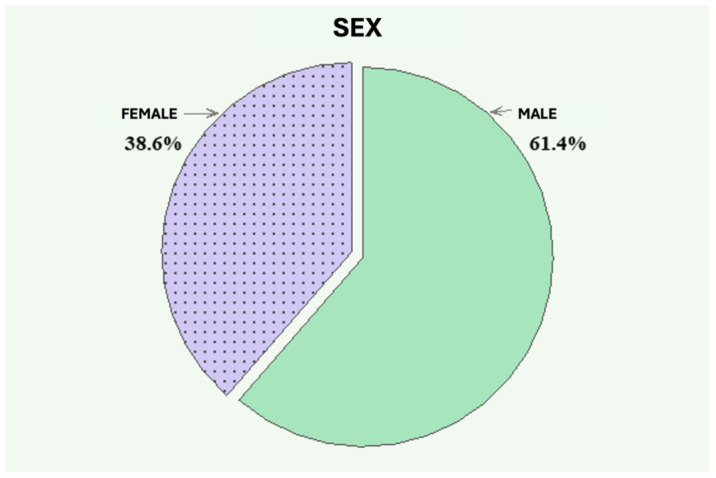
Sex distribution.

This study includes data from 166 patients with all forms of cleft lip and/or palate, treated by one surgeon and seen in consultation to adult or adolescent age in the Department of Pediatric Plastic, Reconstructive, and Aesthetic Surgery at Medical University–Plovdiv, for the period from 1997 to 2024. Of these, 102 were male (61.40%), and 64 were female (38.60%) (Figure 1).

### 2.1. Study Design and Setting

A single-center, retrospective, cohort study was conducted on patients with CL/P who underwent primary surgery at the only specialized Cleft Center in Bulgaria. Care at the center follows internationally accepted cleft protocols, with staged lip, palate, alveolar, and orthognathic procedures coordinated by a dedicated multidisciplinary cleft team.

Each patient treated in the center undergoes a preoperative severity assessment before the primary surgery.

An analysis of all surgical procedures performed on each patient throughout the entire treatment continuum up to maturity was conducted. The number of surgeries for each patient was then stratified by the preoperative severity, and statistical analysis was performed.

Patients who underwent surgical interventions in other settings were excluded from this study.

### 2.2. Patient Population

The study included patients with all forms of cleft lip and/or palate who were treated in the Department of Pediatric Plastic, Reconstructive, and Aesthetic Surgery at University Hospital “St. George”–Plovdiv between 1997 and 2024. All participants met the eligibility criteria for inclusion in the study.

Inclusion criteria:

- Patients with unilateral/bilateral cleft lip ± cleft of the alveolar ridge ± cleft of the palate.
- Nonsyndromic clefts only.
- Patients who were treated only in the Cleft Care Center, without operations performed in another clinic.
- Age—14 years or older.
- Signed consent form.
- Photographic documentation and accompanying medical records.

A total of 166 patients met all criteria and were included in the analysis.

### 2.3. Cleft Classification and Preoperative Severity Grading

**Figure 2:**
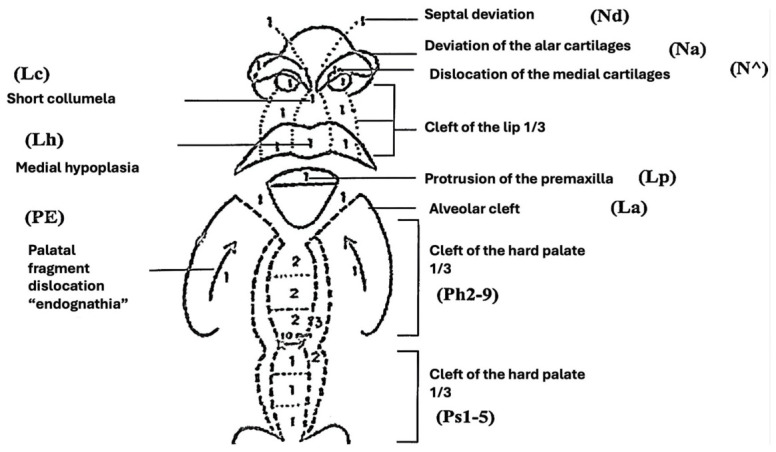
Preoperative severity evaluation graphic Adapted with permission from Ref. [23] (Lc—lip collumela; Lh—lip hypoplasia; PE—palate endognathia; Nd—nose deviation; Na—nose alar cartilages; N^—nose medial cartilages; Lp—lip premaxilla; La—lip alveolar; Ph—palate hard; Ps—palate soft.

The assessment of preoperative severity is an important step in the overall treatment of CLP, as it provides a preliminary plan for the selection of the most appropriate surgical technique for the correction of facial anomalies. The system was introduced by Prof. Y. Anastassov [23,24] and aims to differentiate patients not only by diagnosis and cleft type—unilateral, bilateral—but also by the defect area and associated deformations. The system was also used in studies by other authors [25,26,27,28,29]. A point-based system is used to evaluate the type and form of the defect, depending on whether it affects only the lip; the lip and alveolus; or the lip, alveolus, and palate. Separately, the cleft lip is assessed by microform, incomplete form, and complete form (Figure 2).

Depending on the total score, patients are divided into 4 groups by severity (Table 1).

The system also includes a second postoperative scale that grades the results based on defects that are present after surgery.

Based on the accumulated points, the postoperative result is graded into one of five groups (Table 2).

**Figure 3:**
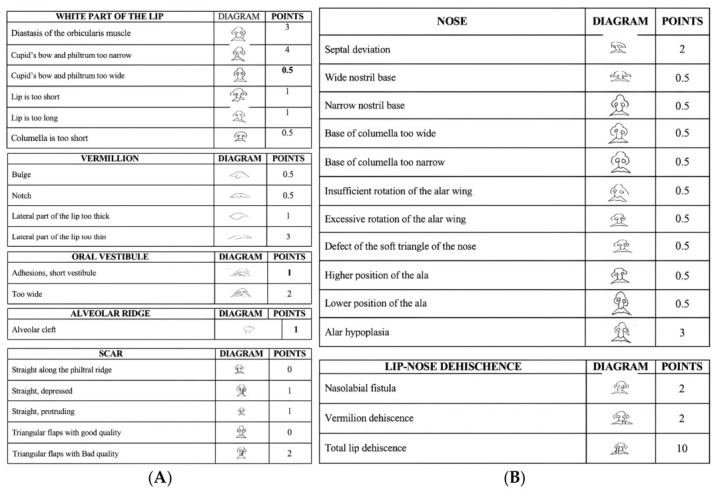
(**A**) Postoperative evaluation rating scales of the lip area. (**B**) Postoperative evaluation of the nose area. Adapted with permission from Ref. [23].

Postoperative evaluation scales play a key role in the decision to perform secondary surgical procedures. The type and severity of the residual defect determines the type of secondary surgery needed (Figure 3). The decision for secondary surgery is made through shared decision-making. For surgical interventions like GPP with alveolar bone grafting, the indications are well defined—a bone defect in the alveolar cleft region mandates such surgery. For surgeries that are more on the cosmetic side—a small lip revision or rhinoplasty—the decision is based on the postoperative evaluation and patients’ opinion.

Inter-rater agreement: Two specialists independently evaluated the preoperative severity of 55 randomly selected patients. Both assessments were made on ordinal scales as follows:

- Preoperative severity: mild, moderate, severe, and very severe.
- Postoperative outcome: very poor, poor, average, very good, and excellent.

To determine the level of agreement between the two raters, the Kappa test was used. The results are summarized in Table 3. Regarding preoperative severity, the analysis showed a high level of agreement of 97.50% according to the Weighted Kappa coefficient (Weighted Kappa = 0.975). A similarly high level of agreement was found in the evaluation of postoperative outcomes—95.20%. Discrepancies between the two raters were few and differed by only one category. For example, Rater 1 assessed the preoperative severity as severe, whereas Rater 2 assessed it as very severe. Disagreements were discussed and resolved with the help of a third specialist and reflected in the final evaluation.

**Figure 4:**
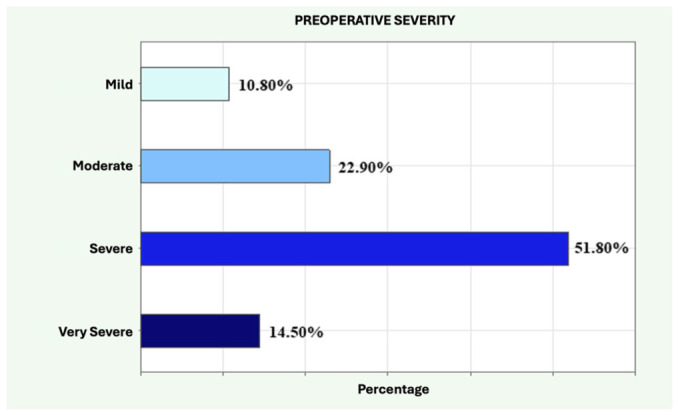
Patient distribution by preoperative severity.

All patients were assigned into one of four groups: Mild, Moderate, Severe and Very Severe. The distribution is shown on Figure 4. 

### 2.4. Surgical Technique

**Figure 5:**
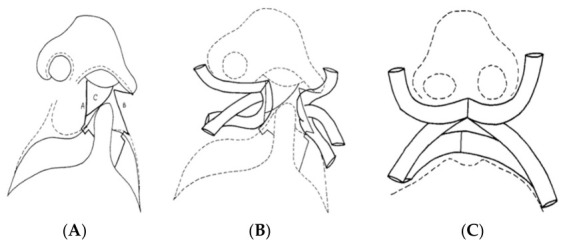
Schematic representation of the modified Millard technique–unilateral. (**A**) mucoperiosteal flap; (**B**) mucoperiosteal flap; (**C**) flap for the reconstruction of the nostril sill. Adapted with permission from Ref [23].

The surgical treatment principles adopted at our center are based on the functional principles of Delaire [30], which were later developed by Professor Philippe Pellerin [31]. The technique incorporates Delaire’s functional principles, esthetic elements from Onizuka [32], but is mainly a modification of Millard’s technique [33,34,35,36,37,38]. The difference is that no “cut back” is performed at the base of the columella with the “C” flap. The incisions and dissections are entirely mucoperiosteal, with no subcutaneous dissection of the muscle, aiming to preserve the philtrum relief. The “C” and “B” flaps are periosteal, aiming to form bone in the floor of the nostril—the upper part of the piriform aperture. Regarding the musculature—the musculus transversus nasi, which inserts into the anterior nasal spine, is dissected in order to reposition the nasal ala medially and vertically. The length of the “C” flap determines the nostril width and provides tissue for forming the columellar base, improving conditions for nasal growth. As for the upper labial frenulum—it is preserved in all cases, as it is believed to stimulate columella growth. At the mucocutaneous border, a “roll flap” is performed to restore the relief in this area and to more precisely approximate both parts of the upper lip. The “C” flap is inserted into an incision made at the level of the alar fold, which is a personal modification. In bilateral forms, the prolabium is not raised, as we preserve the frenulum of the upper lip. The vermilion of the prolabium in bilateral forms is not sacrificed, as it is in Millard’s original technique and bilateral roll flaps are also performed (Figure 5). As for the suture material, a non-absorbable monofilament 4/0 is used for the muscles, and an absorbable braided 4/0 is used for the mucosa. For skin sutures, an absorbable monofilament 6/0 is used. Currently, the technique does not include dissection of nasal anatomy—septum and alar cartilages. The technique does not include primary dissection of nasal structures—specifically the septum and alar cartilages—as the long-term risks of surgical trauma to central facial structures are considered to outweigh the potential benefits. No orthopedic devices or plates were used for preoperative orthopedic or orthodontic preparation or nasoalveolar molding.

For this study, surgical interventions were categorized as follows:

- Primary surgery: The initial cheiloplasty, with or without concurrent primary gingivoperiosteoplasty (GPP). GPP is performed concurrently with primary lip repair when the alveolar cleft width is small, and the position of the medial and lateral fragments shows no significant discrepancy. The technique consists of elevation of the periosteum without cut-back. When the inter-fragment distance exceeds 4 mm or significant dislocation is present, GPP is performed during palate surgery in most cases.
- Secondary or corrective surgeries: Any subsequent cleft-related operation on the lip, nose, or alveolus, including corrective cheiloplasty, GPP with bone grafting, GPP without grafting, rhinoplasty, and corrective rhinoplasty.

Procedures performed for non-cleft indications under general anesthesia were not included.

### 2.5. Statistical Methods

Frequency analysis was used to determine the number and relative proportion of patients by type of primary operation and subsequent surgical interventions. Results were illustrated with pie charts and bar graphs.

The relationship between corrective surgical interventions involving the oral and nasal regions during the treatment continuum and the severity of cleft deformity was assessed using the Kruskal–Wallis test and pairwise post hoc analysis with Bonferroni correction, including six comparisons: (1) mild vs. moderate, (2) mild vs. severe, (3) mild vs. very severe, (4) moderate vs. severe, (5) moderate vs. very severe, and (6) severe vs. very severe. Box plots of medians, interquartile ranges, and individual value distributions are presented.

For data analysis and graphical presentation, the following statistical software was used:

- IBM Corp. Released 2020. IBM SPSS Statistics for Windows, Version 27.0. IBM Corp, Armonk, NY, USA.
- Minitab 22.1 Statistical Software (2024). [Computer software]. Minitab, Inc., State College, PA, USA.

## 3. Results

All 166 patients underwent a primary surgical intervention, with 86 (51.80%) undergoing cheiloplasty and the remaining 80 (48.20%) undergoing cheiloplasty with GPP.

**Figure 6:**
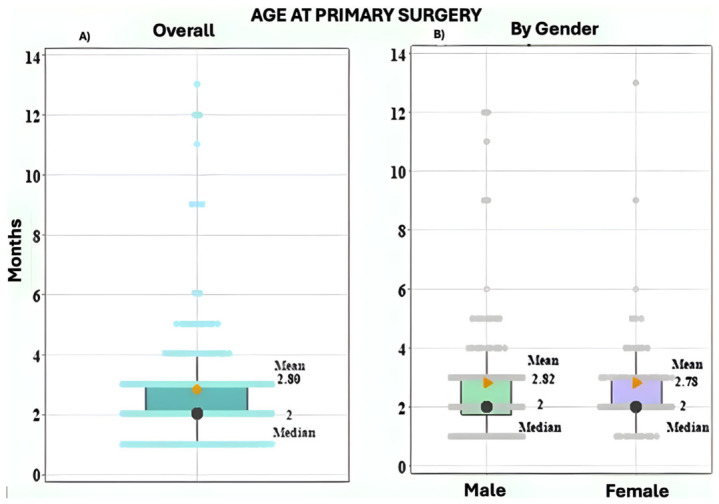
Mean and median age: (**A**) overall; (**B**) based on gender.

The age of the patients at the time of primary lip surgery ranged from 1 to 13 months, with a median of 2 months and a mean of 2.80 ± 2.05 months. Among males, the average age for primary surgery was 2.82 ± 2.16 months compared with 2.78 ± 1.88 months for females, with no significant difference (*p* = 0.898). In both sexes, the median age was 2 months, as was the case for the entire group. Figure 6 presents box plots illustrating the individual and mean age for the whole group (panel A) and by sex (panel B).

**Figure 7:**
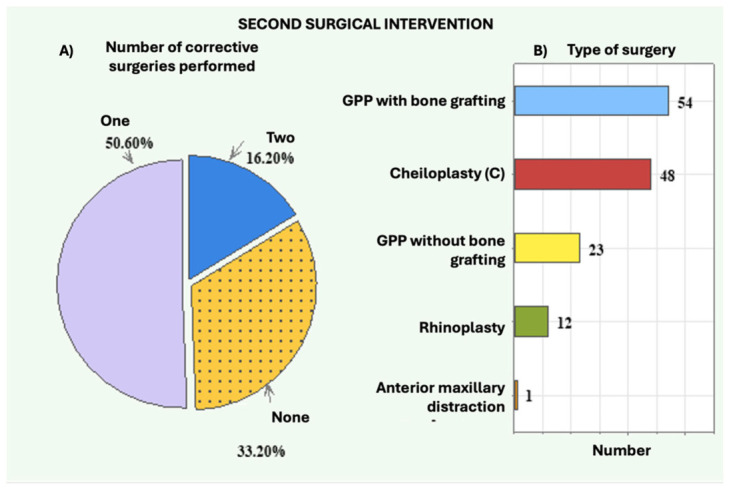
(**A**) Distribution of a second surgical intervention; (**B**) Type of the surgical procedure. C—corrective. GPP—gingivoperiosteoplasty. One—one corrective surgery. Two—two corrective surgeries during the same surgical intervention.

A second surgical intervention was performed on 111 (66.80%) patients. Of these, 84 underwent one operation and 27 underwent two operations in a single stage, bringing the total number of secondary surgical procedures to 138. Patients who underwent a second surgical procedure were between the ages of 1 and 26 years, with a median age of 7 years and a mean of 8.29 ± 4.72 years. According to the type of secondary surgical intervention, the most frequent procedure was GPP with bone grafting (*n* = 54). Corrective cheiloplasty was performed on 48 patients, GPP without bone grafting on 23 patients, rhinoplasty on 12 patients, and anterior maxillary distraction on one patient (Figure 7).

**Figure 8:**
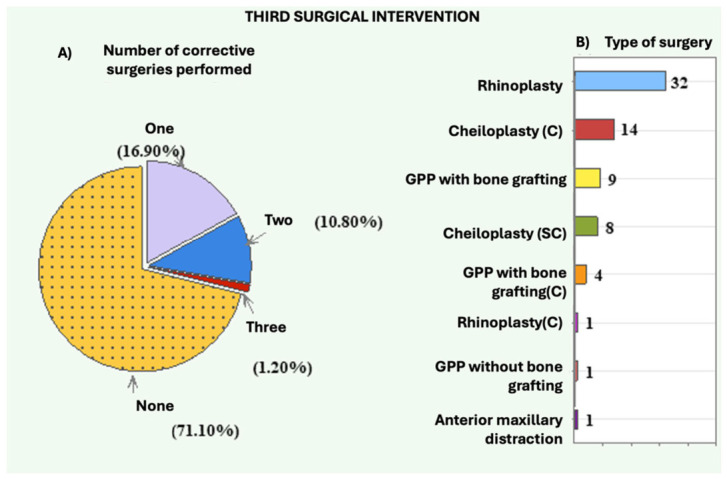
(**A**) Distribution of a third surgical intervention. (**B**) Type of surgical procedure. C—corrective, SC—second corrective. GPP—gingivoperiosteoplasty. One—one corrective surgery. Two—two corrective surgeries during the same surgical intervention. Three—three corrective surgeries during the same surgical intervention.

A third surgical intervention was performed on 48 (28.90%) patients aged between 5 and 27 years, with a median age of 15 years and a mean of 15.25 ± 3.87 years. Of the 48 patients, 28 (16.90%) underwent one corrective surgery, 18 (10.80%) underwent two corrective surgeries, and two (1.20%) underwent three corrective surgeries. The total number of corrective surgeries was 70, with the following distribution by type: 32 rhinoplasties, 14 corrective cheiloplasties, nine GPP with bone grafting, eight corrective cheiloplasties, four corrective GPP with bone grafting, one GPP without bone grafting, and one corrective rhinoplasty (Figure 8).

**Figure 9:**
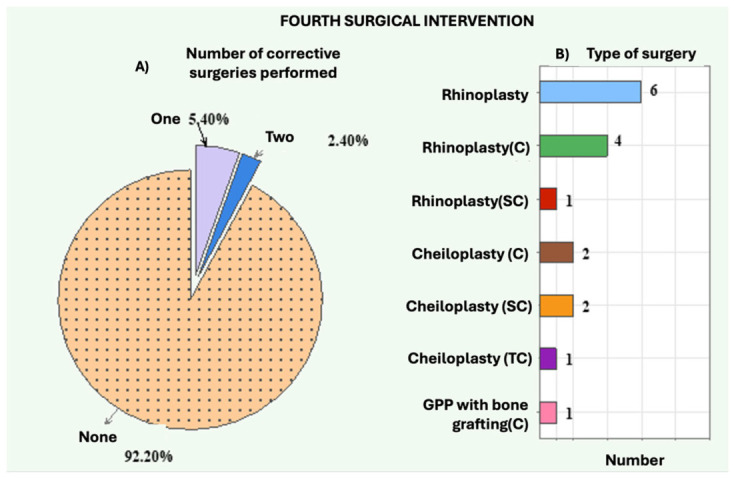
(**A**) Distribution of a fourth surgical procedure. (**B**) Type of the surgical procedure. C—Corrective, SC—second corrective. TC—third corrective. GPP—gingivoperiosteoplasty. One—one corrective surgery. Two—two corrective surgeries during the same surgical intervention.

A fourth surgical intervention was performed on 13 (7.80%) patients aged between 13 and 23 years, with a median age of 16 years and a mean of 16.69 ± 3.22 years. Of these, nine (5.40%) underwent one corrective surgery and four (2.40%) underwent two corrective surgeries, bringing the total number of corrective surgeries to 17. Rhinoplasty was performed on six patients, corrective rhinoplasty on four patients, and second corrective rhinoplasty on one patient. Corrective cheiloplasty was performed on two patients, second corrective cheiloplasty on two patients, and third corrective cheiloplasty on one patient. One patient underwent corrective GPP with bone grafting (Figure 9).

**Figure 10:**
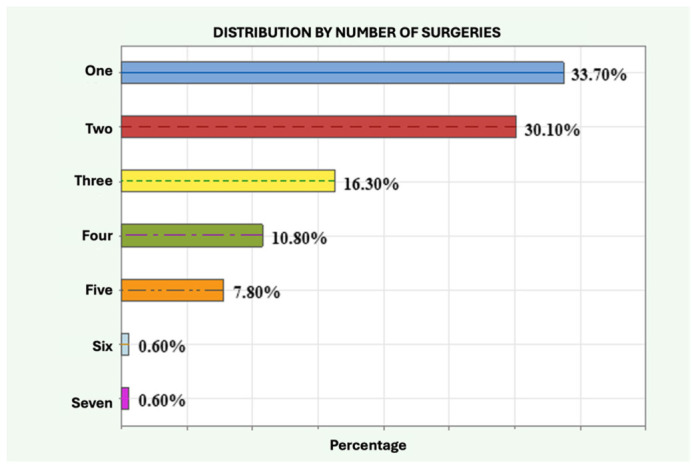
Distribution of the patients based on the number of surgical corrections.

According to the total number of surgical interventions, including the primary and all subsequent corrective operations during the treatment continuum, the patients were divided as follows: 56 (33.70%) patients underwent one primary surgery; 50 (30.10%) patients underwent two surgical procedures—one primary and one corrective surgery; 27 (16.30%) patients underwent three surgical procedures—one primary and two corrective surgeries; 18 (10.80%) patients underwent four surgical procedures—one primary and three corrective surgeries; 13 (7.80%) patients underwent five surgical procedures—one primary and four corrective surgeries; and one (0.60%) patient underwent six surgical procedures—one primary and five corrective surgeries (Figure 10).

These data indicate that approximately two-thirds of patients required at least one secondary procedure and nearly one-fifth underwent four or more surgeries. The pattern of early alveolar procedures followed by later nasal and lip revisions is consistent with published long-term treatment series in complete BCLP and CLA [10,11,12,13,14,15,16].

The relationship between the total number of surgical interventions, including the primary and all subsequent corrective operations, and the preoperative cleft severity was examined using the Kruskal–Wallis test and post hoc pairwise analysis with the Bonferroni test, which included six comparisons: (1) mild vs. moderate, (2) mild vs. severe, (3) mild vs. very severe, (4) moderate vs. severe, (5) moderate vs. very severe, and (6) severe vs. very severe. The results of the statistical analysis are presented in Table 4. A significant relationship between the two variables was found using the Kruskal–Wallis test (*p* < 0.001). The pairwise comparisons revealed the following significant differences. Patients with mild severity had significantly fewer surgical interventions than those with severe severity (*p* < 0.001) and very severe severity (*p* = 0.02). The number of surgical interventions in patients with moderate severity was also significantly lower in those with severe (*p* < 0.001) and very severe severity (*p* = 0.023). The other comparisons—between mild and moderate (*p* = 1.000) and between severe and very severe (*p* = 0.411)—did not reveal significant differences.

**Figure 11:**
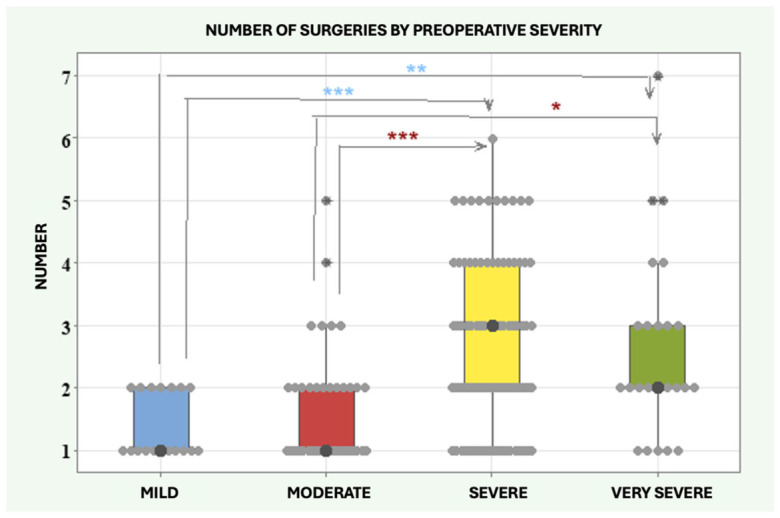
Boxplots of the number of surgical interventions in relation to the preoperative severity. *—statistically significant when *p* < 0.05; **—statistically significant when *p* < 0.01; ***—statistically significant when *p* < 0.001.

Figure 11 presents boxplots of the number of surgical interventions, including the primary operation and all subsequent corrective procedures, relative to patient severity. The figure illustrates medians, interquartile ranges, and distributions of individual values.

Thus, patients with severe or very severe preoperative deformities experienced a substantially higher surgical burden than those with mild or moderate deformities, whereas differences within the mild–moderate and severe–very severe pairs were not statistically significant. Boxplots of total surgeries by severity grade visually demonstrate this pattern, with median values clustering around a single operation for mild and moderate deformities and higher medians and broader distributions for severe and very severe clefts.

## 4. Discussion

This single-center study confirms that patients with cleft lip, followed to a mean age of 18 years, experience a considerable long-term surgical burden. Only one-third of patients completed their treatment with a single primary operation, while two-thirds underwent at least one additional procedure and nearly one-fifth required four or more operations on the lip, nose, or alveolus. The timing and nature of these interventions—early GPP/bone grafting followed by adolescent and young adult rhinoplasty and lip revisions—mirror typical cleft care pathways described in long-term series [10,11,12,13,14,15,16].

A key and novel observation is the strong association between preoperative nasolabial severity and cumulative surgical burden. Mild and moderate deformities generally required only one or two operations, whereas severe and very severe clefts had significantly higher total surgery counts. This finding is congruent with the hypothesis that more distorted anatomy at baseline is harder to correct definitively and more likely to necessitate staged revisions as the patient grows and functional and esthetic expectations evolve [10,11,12,13,14,15,16,21,22].

Although this study demonstrates a clear step-up in surgical burden when moving from mild–moderate to severe–very severe deformities, there is still notable variability in the number of operations among patients within each severity category. Several factors likely contribute to this heterogeneity and are important to acknowledge when interpreting the results.

A notable methodological strength is the absence of inter-surgeon variability, as all operations were performed by a single surgeon. Patient and family preferences can strongly shape the number and type of later procedures. Families differ in their tolerance for additional surgery, their willingness to accept residual asymmetry or scarring, and their aspirations regarding appearance. Adolescents and young adults may have changing priorities as they mature—some may actively seek further refinement for psychosocial reasons, while others decline surgery despite clear anatomical indications. These preference-sensitive decisions can lead to divergent surgical paths for patients with similar preoperative severity.

Another important reason could be that growth patterns and biological variability also introduce considerable unpredictability. Craniofacial growth is not uniform, and small differences in maxillary growth, scar behavior, nasal cartilage development, or dental eruption can compound over time. Two patients with the same preoperative severity may diverge substantially in midfacial projection or dental arch form, leading to different functional needs and esthetic concerns, and consequently different indications for secondary surgery.

Together, these considerations suggest that while preoperative severity is a powerful predictor at the group level, it cannot fully determine individual treatment trajectories. Any severity-based prognostic counseling should therefore be presented as a range or probability rather than as a fixed prediction for a given patient.

The observed association between higher severity and greater surgical burden naturally raises the question of what constitutes an effective or successful treatment pathway in cleft lip care. Counting operations is informative, but it is an incomplete proxy for both burden and efficacy. More surgery is not necessarily “worse” if it delivers clear functional and psychosocial benefits; fewer operations are not necessarily “better” if they leave patients with avoidable problems that impair their quality of life.

From a narrow procedural perspective, an “efficient” protocol would achieve acceptable function and appearance with the fewest possible operations and anesthetic exposures. This perspective motivates efforts to refine primary techniques, optimize timing, and reduce the need for revision, especially in high-severity cases. However, from a patient-centered perspective, the goal is not simply to minimize interventions but to maximize long-term well-being. For some individuals, an additional rhinoplasty or lip revision in late adolescence may significantly improve self-image, social confidence, or professional opportunities and thus represent a highly efficient use of surgical resources despite adding to the numerical burden.

There is also a philosophical tension between normalization and acceptance. Modern cleft surgery seeks to restore facial form as close to “normal” as possible, but there are limitations to what surgery can achieve, especially in the context of severe deformities and complex growth patterns. At some point, the incremental esthetic gains of further surgeries may diminish, while the physical, psychological, and economic costs remain. Deciding where that point lies is inherently value-laden and must be negotiated between clinicians, patients, and families. For some, accepting a degree of residual asymmetry may be associated with better psychosocial adjustment. For others, pursuing an additional operation may meaningfully improve life satisfaction.

In this light, the present findings do not imply that reducing the number of surgeries should be an absolute goal. Rather, they suggest that preoperative severity can help identify patients in whom clinicians should be particularly deliberate about the trade-offs between additional procedures and expected benefit, ensuring that each operation has a clear and meaningful purpose. Ultimately, truly effective cleft care will be defined not by the shape of the surgical count distribution but by whether children born with cleft lip can enter adulthood with good function, acceptable appearance, and a sense of agency and satisfaction regarding the care they received.

Prior studies have quantified the total number of surgeries for patients with CL ± P from birth to late adolescence or early adulthood. McIntyre et al. reported a mean of 8.6 procedures (SD 4.4) and 6.7 anesthesia events in a cohort of 71 patients, with higher surgical loads in those with more extensive clefts. Janssen et al. found that within the first six years of life, children with unilateral CLP treated at accredited cleft centers had significantly fewer cleft operations than those managed in non-accredited centers. A recent Canadian study documented a median of 10.5 surgical procedures and 148.5 healthcare interactions from 0 to 18 years in nonsyndromic CLP patients, underlining the extensive burden of care even in high-resource settings [7,8,39].

The overall distribution in the present cohort—many patients with one to three operations and a substantial minority with four or more—is at the lower end of the ranges reported in these series, possibly reflecting a relatively standardized protocol and concentrated care in a dedicated cleft unit [3,4,7,8,9,10,20]. Longitudinal cohort studies of complete BCLP and CLA have similarly documented multiple staged procedures over two decades, including primary lip and palate repair, several secondary lip/nose surgeries, velopharyngeal operations, alveolar bone grafting, and orthognathic surgery [10,11,12,13,14,15,16,17,21,22]. For example, Hattori et al. reported an average of 5.9 operations per patient with complete BCLP, with 64.8% undergoing revisional lip/nose surgery after skeletal maturity and 60.7% requiring orthognathic surgery. In patients with complete CL, the same group found that bilateral cases had more operations and a higher likelihood of repeated alveolar grafting than unilateral cases [9,34].

The current study adds to this literature by explicitly linking preoperative severity grading to surgery counts. The Unilateral Cleft Lip Severity Index and related scales, validated by Campbell et al., have shown that worse baseline deformity predicts more challenging reconstructions and a greater need for secondary revision. However, few reports have quantified total surgery numbers across severity strata into late adolescence. The present findings—higher median and mean surgery count in severe and very severe clefts compared with mild and moderate—support the prognostic relevance of systematic severity assessment and are consistent with trends seen in long-term BCLP and CL cohorts [1,2,10,11,12,13,21,22,40].

Alveolar cleft management contributes substantially to the overall surgical load. Gingivoperiosteoplasty (GPP) and secondary alveolar bone grafting (SABG) are the main options, but their long-term impacts on the need for additional bone grafts, orthodontic interventions, and maxillary growth remain debated [40,41,42,43]. Prospective and retrospective comparative studies have shown that primary GPP can reduce the need for later SABG in some patients but may also be associated with higher rates of residual defects and re-grafting [41,42,43,44]. Systematic reviews of SABG emphasize that bony reconstruction of the alveolar process is a key late step in achieving functional and esthetic rehabilitation but may require reintervention in a proportion of patients [11,12,13]. 

This cohort’s frequent use of primary and secondary GPP, with bone grafting as the most common second-stage procedure and further corrective GPP at the third and fourth stages, underscores the contribution of alveolar reconstruction to overall surgical burden. The pattern observed here—recurrent GPP with bone grafting among patients undergoing multiple interventions—is consistent with these reports and reinforces that alveolar reconstruction strategies can influence long-term surgical load [13,43].

From a patient perspective, qualitative and PROM-based studies, including those using the CLEFT-Q instrument, emphasize that residual nasal asymmetry, lip scarring, dental irregularities, and skeletal discrepancies continue to affect quality of life in adolescence and adulthood [14,15,16,18,19,20,44]. Sinko et al. and others have demonstrated that late revisions and orthognathic surgery can significantly improve functional and psychosocial outcomes in adults with CLP [18,19,39]. The predominance of rhinoplasty and lip revisions among third and fourth interventions in this cohort aligns with these observations, illustrating how long-term psychosocial and esthetic goals drive continued demand for surgery.

These data provide severity-stratified, long-term information that can enhance counseling at the time of primary repair. For mild and moderate clefts, clinicians can typically advise that one or two surgeries are often sufficient for satisfactory outcomes, while acknowledging that some patients may still desire or need further refinement [7,8,9,21,22,23]. In contrast, families of children with severe or very severe deformities should be prepared for a high likelihood of multiple staged procedures, including adolescent rhinoplasty, lip revision, and potentially repeated alveolar interventions [10,11,12,13,16,17,18]. Such transparent counseling can align expectations with realistic trajectories and support shared decision-making [8,9,10,18,19,20].

A central finding of this study is the clear association between preoperative nasolabial severity and long-term surgical burden. Patients with mild or moderate deformities generally required only one or two operations, whereas those classified as severe or very severe underwent significantly more procedures over the course of their treatment. This step-change in surgical burden between the lower- and higher-severity groups suggests that preoperative severity grading captures clinically meaningful differences in treatment complexity that persist over many years. At the same time, the lack of a statistically significant difference between mild and moderate, and between severe and very severe, indicates that beyond certain thresholds, incremental changes in severity do not translate into further increases in surgery counts.

### 4.1. Strengths and Limitations

Strengths of this study include the relatively long follow-up (mean age 18 years), allowing capture of major childhood and adolescent revisions; the use of a structured, clinically grounded four-level severity scale; and the focus on a clear, tangible primary endpoint (total number of surgeries) that is easily interpretable and comparable to other burden-of-care studies [7,8,9,10,21,22]. The single-center setting provides consistency of surgical protocols and follow-up routines.

Limitations include the retrospective design and reliance on the completeness of medical records. The severity grading system, while informed by validated indices, is not identical to existing scales and may limit direct comparability across centers. A further limitation is the measurement of surgical burden solely as a procedure count. Important dimensions such as procedure complexity, operative time, anesthesia exposure, complications, costs, and psychosocial impact are not addressed.

Patient and family preference for revision surgery, as well as socioeconomic status, represent uncontrolled confounding factors that may influence the observed surgical counts. The single-center, single-surgeon context, while ensuring internal consistency, limits external validity, as differences in surgical philosophy, esthetic standards, and decision-making for secondary procedures may affect generalizability to other settings. Finally, the study focused on surgery counts and did not incorporate functional outcomes, growth data, or PROMs, which are crucial for evaluating the value rather than just the quantity of interventions [1,2,18,19,20,21].

### 4.2. Future Directions

Future research should integrate preoperative severity with multidimensional outcome measures, including validated esthetic assessments and PROMs, such as the CLEFT-Q [18,19,20,21,45,46]. Multicenter, prospective cohorts using standardized severity indices and harmonized treatment protocols would allow more robust modeling of how specific surgical decisions at each stage—choice of primary technique, use of NAM, timing and type of alveolar reconstruction, and approach to orthognathic correction—influence both outcome quality and long-term surgical burden across different severity strata [2,3,4,6,7,8,9,10,23]. Ultimately, severity-adjusted algorithms could be developed to optimize the balance between outcome excellence and minimized lifetime treatment burden.

Treatment efficiency—defined as the number of surgeries required to achieve very good or excellent outcomes—and follow-up quality, assessed by evaluating patients at key growth milestones through full adulthood, should be considered as candidate quality indicators for good clinical practice and protocol benchmarking and could serve as metrics for evaluating the performance of a National Reference Center. The preoperative severity scale and the postoperative outcome scale for the residual defects and surgical outcomes have been integrated into our electronic medical record from 2013, and statistical data can be obtained on a real-time basis [28,44,47].

## 5. Conclusions

In this cohort of 166 patients with cleft lip followed to a mean age of 18 years, the majority required more than one cleft-related operation, and a substantial minority underwent four or more procedures on the lip, nose, or alveolus. Preoperative severity of the nasolabial deformity was a strong predictor of this long-term surgical burden: patients with severe or very severe clefts underwent significantly more surgeries than those with mild or moderate deformities. The temporal pattern of these interventions—earlier alveolar and lip procedures in childhood followed by nasal and lip refinements in adolescence and early adulthood—illustrates the longitudinal nature of cleft care. These findings have direct implications for clinical practice. First, they support the systematic use of preoperative severity assessment as a prognostic tool. Discussing likely surgical trajectories in a severity-stratified way can help clinicians provide more accurate counseling to families at the time of primary repair, aligning expectations with realistic long-term outcomes. Second, the concentration of high surgical burden in the severe and very severe groups identified a target population for protocol refinement, such as enhanced pre-surgical orthopedics, more comprehensive primary nasolabial correction, or carefully staged alveolar strategies, with the explicit aim of reducing the need for multiple later revisions.

Finally, this study highlights both the potential and the limits of using surgery counts alone as a measure of burden of care. A higher number of operations may reflect greater anatomical complexity, evolving esthetic expectations, or a deliberate strategy of staged refinement rather than suboptimal primary surgery. Future research should therefore integrate severity-based surgical burden data with functional outcomes and patient-reported measures to distinguish necessary and beneficial interventions from potentially avoidable procedures. In summary, preoperative cleft lip severity is a robust predictor of long-term surgical burden in this cohort, and incorporating structured severity assessment into routine practice can enhance prognostication, inform protocol development, and ultimately contribute to more patient-centered and efficient cleft care.

## Figures and Tables

**Table 1:** Groups of preoperative severity.

Groups	Lip–Nose	Palate	Overall
Mild	1–3	1–3	1–4
Moderate	4–6	4–10	5–12
Severe	7–11	11–13	13–21
Very Severe	12–16	14–16	22–32

**Table 2:** Groups of postoperative results.

Groups	Lip–Nose	Palate	Overall
Excellent	0–1.5	0–1.5	0–2
Very Good	2–3.5	2–3.5	2.5–6.5
Good	4–5.5	5.5–7	7–10
Bad	6–8	7.5–8	1.5–16
Very Bad	Over 8.5	Over 8.5	Over 16.5

**Table 3:** Interrater agreement for the preoperative severity and postoperative results.

	Kappa		Kappa Weighted		
Measurement	Coefficient	95% CI	Coefficient	95% CI	
Preoperative severity	0.963	0.892–1.000	0.975	0.926	1.000
Postoperative result	0.938	0.845–1.000	0.952	0.883	1.000

95% CI—95% confidence interval.

**Table 4:** Correlation between preoperative severity and the number of surgeries.

Statistics	Mild (1)	Moderate (2)	Severe (3)	Very Severe (4)	Kruskal–Wallis *p*	Bonferroni *p*
Median (IQR)	1 (1)	1 (1)	3 (2)	2 (1)	<0.001	1↔2: 1.000 1↔3: <0.001 1↔4: 0.002 2↔3: <0.001 2↔4: 0.023 3↔4: 0.411
Mean (SD)	1.38 (0.50)	1.71 (0.95)	2.72 (1.36)	2.62 (1.49)		

IQR—interquartile range; SD—standard deviation.

## Data Availability

All data are available upon request from the authors.

## Abbreviations

The following abbreviations are used in this manuscript:

CL	Cleft Lip
CL ± P/CLP	Cleft Lip with or without Cleft Palate
CLA	Complete Cleft Lip and Alveolus
BCLP	Complete Bilateral Cleft Lip and Palate
GPP	Gingivoperiosteoplasty
SABG	Secondary Alveolar Bone Grafting
PROMs	Patient-Reported Outcome Measures
CLEFT-Q	A validated patient-reported outcome instrument for cleft lip/palate
SD	Standard Deviation
IQR	Interquartile Range
CI/95% CI	Confidence Interval/95% Confidence Interval
IBM SPSS	IBM Statistical Package for the Social Sciences
B/C/SC/TC	Corrective surgery indicators in figures (C—Corrective; SC—Second Corrective, TC—Third Corrective)
NAM	Nasoalveolar Molding
